# The Multidisciplinary Management of A Fusion Involving a Permanent Maxillary Central Incisor and a Partially Erupted Supernumerary Tooth: A Case Report

**DOI:** 10.1002/ccr3.73560

**Published:** 2026-09-20

**Authors:** Andrew Irvine, Saarah Juman, John Marley, Patrick Fee, Alistair McNeill, Chris Johnston

**Affiliations:** ^1^ Dental Core Trainee, School of Dentistry Royal Victoria Hospital Belfast Northern Ireland; ^2^ Post‐CCST, Orthodontic Department, School of Dentistry Royal Victoria Hospital Belfast Northern Ireland; ^3^ Oral Surgery Consultant, Oral Surgery Department, School of Dentistry Royal Victoria Hospital Belfast Northern Ireland; ^4^ Restorative Dentistry Consultant, Restorative Department, School of Dentistry Royal Victoria Hospital Belfast Northern Ireland; ^5^ Orthodontic Consultant, Orthodontic Department, School of Dentistry Royal Victoria Hospital Belfast Northern Ireland

**Keywords:** cone‐beam computed tomography, fused teeth, incisor, supernumerary, tooth

## Abstract

A 12‐year‐old female patient was initially referred to and examined in the oral surgery department at the School of Dentistry, Belfast following referral from her high street orthodontist. Reason for referral was extraction of two supernumeraries associated with the palatal aspect of the maxillary central incisors along with removal of two retained maxillary primary canines, to facilitate orthodontic treatment with fixed appliances. Although not clinically evident, dental cone beam computed tomography (CBCT) revealed fusion between the right maxillary central incisor and adjacent palatal supernumerary tooth at the enamel level. This discovery prompted a multidisciplinary approach with comprehensive treatment planning, to separate the fusion and remove the supernumerary tooth. Pre‐surgical input from the orthodontic department focused on fixed appliance placement to support the splinting of the maxillary central incisor, providing stability intra‐operatively and during the post‐operative healing period. Consultation with the restorative department centered on contingency planning, should the central incisor be lost during surgery, including fabrication of a removable prosthesis and discussion of definitive replacement options. This culminated in successful surgical separation of the fusion and removal of the supernumerary teeth. This case highlights the value of CBCT in confirming the presence of dental anomalies and demonstrates the importance of multidisciplinary planning in optimizing treatment outcomes whilst minimizing the risk of permanent tooth loss.

## Key Clinical Message

Tooth fusion is the union between the enamel or enamel and dentin of two or more separate tooth germs. This case highlights the multidisciplinary management of a fusion involving a maxillary central incisor and adjacent supernumerary tooth. It covers diagnosis, pre‐operative restorative considerations and orthodontic treatment, followed by surgical separation.

## 1 Introduction

Tooth fusion is defined as the union between the enamel and/or dentin of two or more separate tooth germs [1]. The literatures suggests fusion is more common in the primary dentition than in the permanent dentition, with the incidence rate ranging from 0.5% to 2.5% and from 0.01% to 0.2% respectively [2, 3, 4].

A supernumerary tooth is defined as one that is additional to the normal series [5]. The incidence rate varies in the literature but is usually 0.3%–0.8% in the primary dentition and 1.5%–3.5% in the permanent dentition [6, 7]. According to the literature, the most common location for supernumerary teeth is the anterior maxillary region [8, 9], and this case report involves a patient presenting with two such supernumerary teeth, one of which is fused to the maxillary right central incisor. Most of these supernumeraries are asymptomatic and fail to erupt, only discovered on routine radiographic examination.

Fusions involving a permanent tooth and a supernumerary tooth are an extremely rare occurrence with evidence largely based on isolated case reports and case series. Therefore, this case is best considered in the context of a rare subgroup within an already rare group, with two uncommon developmental anomalies occurring concurrently. In asymptomatic cases, where aesthetics, function and oral hygiene are unaffected, monitoring may be a reasonable option. However, in this case, retention of the fused supernumerary tooth was impeding planned tooth movement. This case report emphasizes the diagnostic value of cone‐beam computed tomography (CBCT) and examines the preoperative multidisciplinary management and surgical separation of the fusion.

## 2 Case History

**FIGURE 1:**
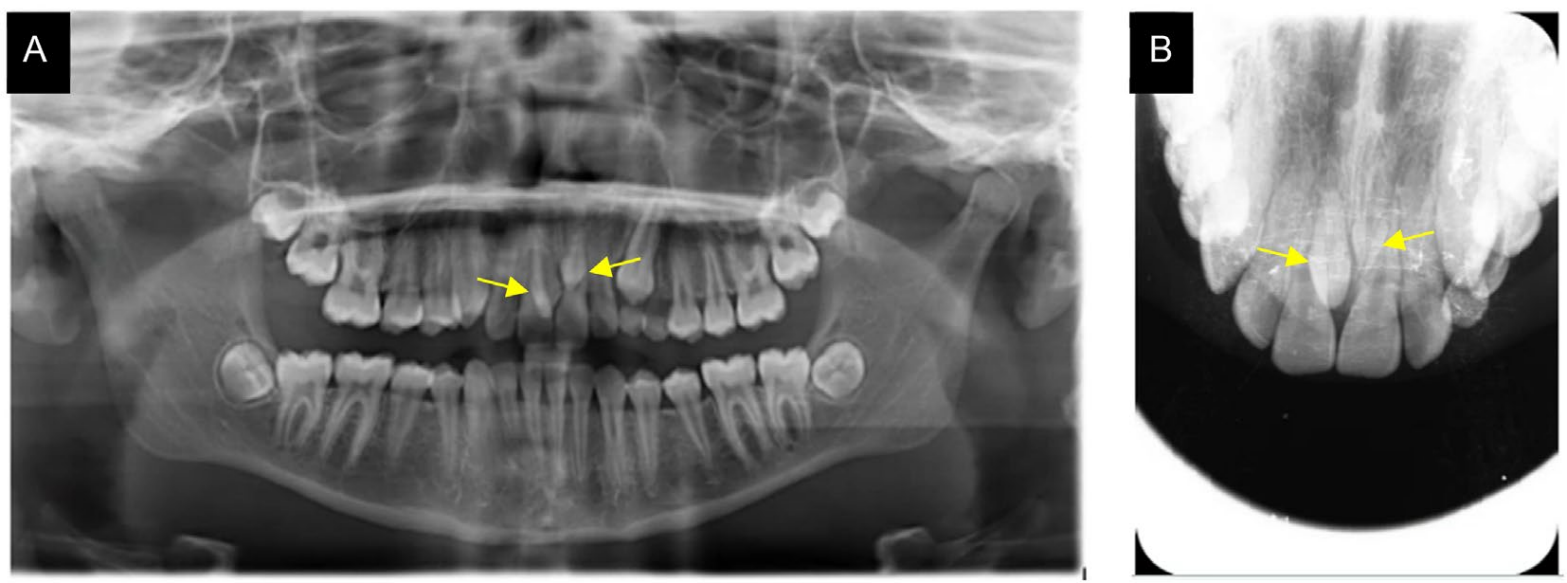
(A) Orthopantomograph showing full permanent dentition with retained maxillary left deciduous teeth (ULC and ULD) and supernumerary teeth present. (B) Upper standard occlusal radiograph showing the maxillary anterior dentition and supernumerary teeth.

A 12‐year‐old female patient was initially examined in the oral surgery department at the School of Dentistry, Belfast in April, 2025 following referral from her high street orthodontist. Her medical history was unremarkable and there was no history of dental trauma or family history of fusion. Reason for referral was extraction of two supernumeraries associated with the palatal aspect of both maxillary central incisors along with removal of two retained maxillary primary canines, to facilitate orthodontic treatment. However, in the interim between referral and initial assessment, the maxillary right primary canine exfoliated naturally, with its successor fully erupted as shown on the radiographs provided by the patient's orthodontist in Figure 1.

**FIGURE 2:**
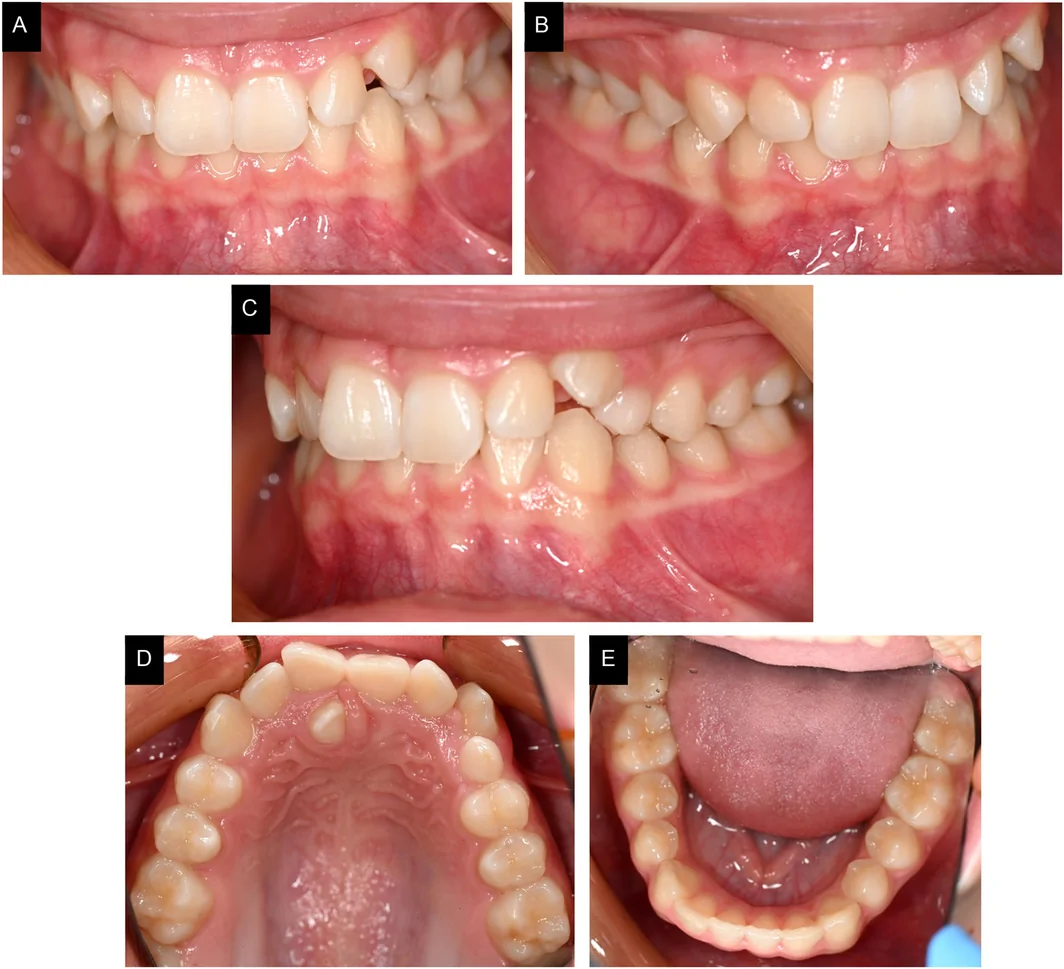
Intraoral photographs showing (A) Frontal (B) Right lateral and (C) Left lateral views of the patient's dentition in occlusion. (D) An intra‐oral view of the maxillary dentition. (E) An intra‐oral view of the mandibular dentition.

On examination, the patient had a well‐maintained dentition, with the right supernumerary tooth partially erupted and lying palatal to the maxillary right central incisor, as shown in Figure 2.

The left supernumerary tooth was unerupted and maxillary left primary canine in situ, with the maxillary left permanent canine partially erupted. The partially erupted supernumerary was first noted 6 months prior to initial attendance and vitality testing of the maxillary incisors were all positive, with no tenderness to percussion noted.

## 3 Investigations

**FIGURE 3:**
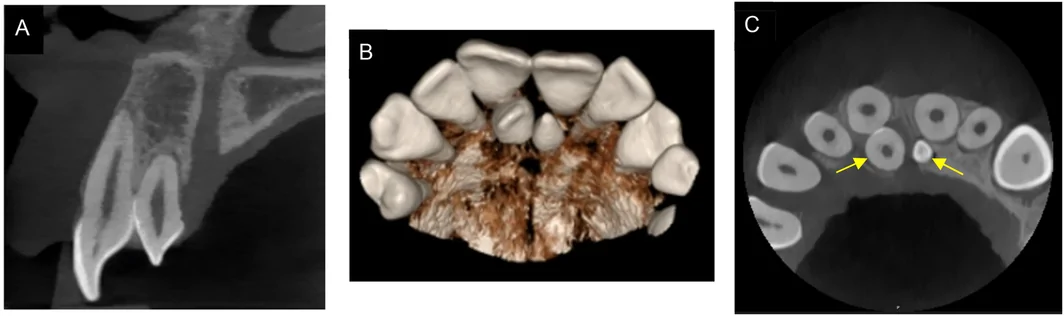
CBCT images showing (A) A sagittal view of the fusion. (B) A 3D view of the fusion and anterior maxillary dentition from the palatal aspect. (C) An axial view of the fusion and anterior maxillary dentition.

A cone‐beam computed tomograph (CBCT) was taken to assess the relationship between the supernumerary teeth and maxillary central incisors, particularly in the case of the upper left supernumerary tooth. The CBCT, as shown in Figure 3, revealed possible fusion between the maxillary right central incisor and adjacent supernumerary tooth at the enamel level, necessitating a formal dental and maxillofacial radiology report from the Leeds Dental Institute.

## 4 Diagnosis

This report confirmed ‘continuity of dental tissue between the roots of UR1 and the supernumerary in the coronal third of the roots, consistent with fusion.’ The root of the partially erupted conical supernumerary was also noted to be ‘sinuous’ in the apical third. The report continued, on the contralateral side, a second conical supernumerary is seen to the left of midline, palatal to UL1. There is no contact between the left supernumerary and the central incisor, however there is no bony separation from the incisive foramen. The root is short and straight. This prompted a multidisciplinary meeting between the oral surgery and orthodontic departments.

## 5 Treatment

Orthodontic examination revealed a class II division 2 malocclusion on a class I skeletal base, an upper dental centerline shift to the right and a partially erupted, buccally placed maxillary left permanent canine, with adequate space to accommodate. Class I buccal segments were noted with the upper right central incisor to upper left lateral incisor noted to be retroclined and lower arch well aligned. Treatment planning highlighted the need for removal of the supernumerary teeth to eliminate their mechanical obstruction to orthodontic tooth movement and avoid potential pathology such as resorption, dentigerous cyst development and damage to adjacent teeth. Furthermore, the presence of the partially erupted and fused supernumerary tooth meant movement of the upper right central incisor would be compromised and there was an increased risk of caries and infection associated. The main treatment objectives were alignment, centerline correction, leveling and overbite reduction.

Options explored with the patient were leaving the supernumeraries in situ with subsequent monitoring, and an awareness of the risk of future pathology and intervention. The other option was fixed appliance placement prior to removal of the supernumeraries, involving surgical separation of the fusion and subsequent orthodontic treatment. Fixed appliance placement would support the splinting of the maxillary right central incisor, providing stability intra‐operatively and during the post‐operative healing period. Risks outlined included immediate loss of the maxillary right central incisor intra‐operatively due to loss of palatal bone support during surgery as well as future loss due to downstream pathology. Potential further treatment in such instances was discussed, including root canal treatment or extraction.

**FIGURE 4:**
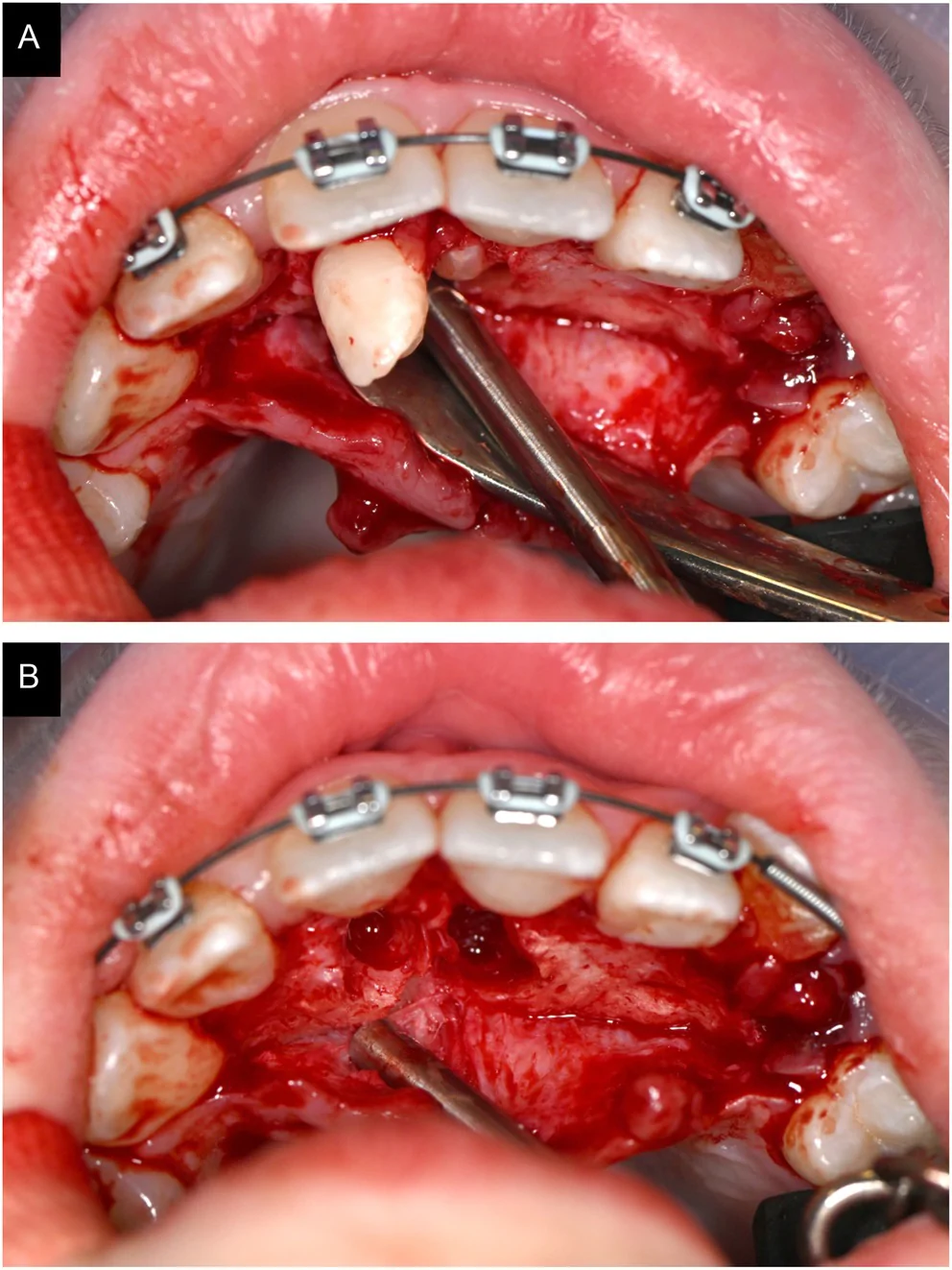
Intra‐operative photographs showing (A) Separation of the fusion, prior to delivery, with part of the unerupted left supernumerary tooth visible as well. (B) The surgical site following removal of the supernumerary teeth with preservation of the maxillary right central incisor.

**FIGURE 5:**
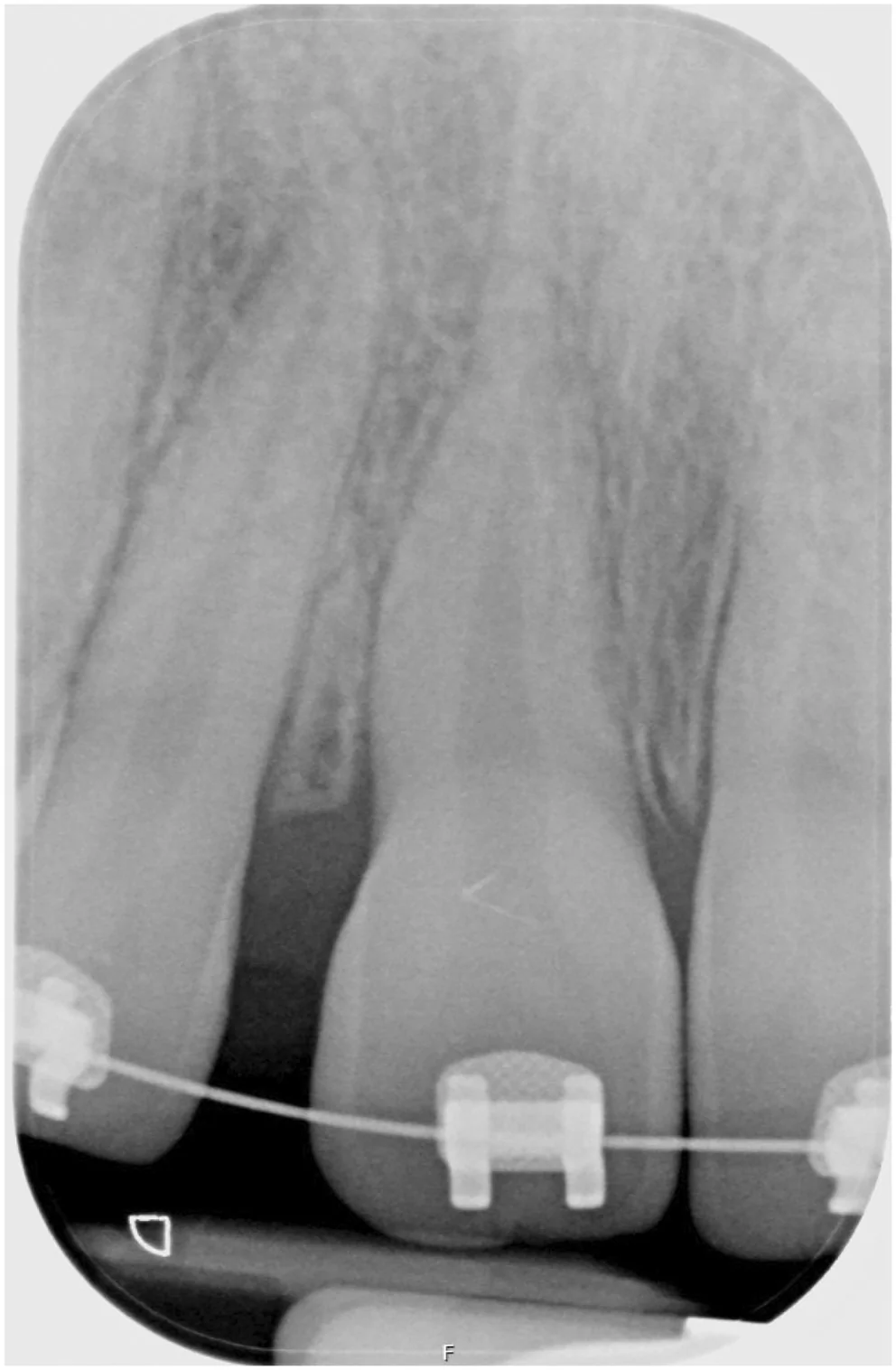
Intra‐oral periapical radiograph of the upper right central incisor taken two months after surgical separation.

Contingency planning was also explored including pre‐operative fabrication of a removable prothesis to replace the maxillary right central incisor should it be lost during the surgery. And then following healing, intermediate to long term contingency planning focused on fixed prosthetic replacement including transitional adhesive bridgework and ultimately implant placement with or without bone grafting when growth has ceased. A high lip line, with 2 mm of maxillary incisor gingiva showing on smiling, raised concerns regarding achievement of an adequate aesthetic result with regards to prosthetic replacement with an adhesive bridge. No periodontal probing depths greater than 3 mm were noted and impressions were taken to construct a removable prosthesis to replace the maxillary right central incisor should it be lost during the surgical separation. The upper fixed appliance was then placed, and a 0.013 nickel titanium wire tied in and later advanced to a 0.018 stainless steel round wire, with the upper left canines left off and upper right premolars partially ligated. In the oral surgery department, separation of the fusion and removal of the supernumeraries and upper left primary canine took place under general anesthetic. Following mucoperiosteal flap reflection, it was confirmed clinically a fusion was in fact present. An osteotome was used to separate the right supernumerary and maxillary right central incisor with digital support of the upper right central incisor. The aforementioned supernumerary was removed intact, with maxillary right central incisor remaining in situ. Intra‐operative photography was completed as shown in Figure 4, documenting the surgical procedure, and post‐operative review revealed the patient to be healing well with retention of vitality and no radiographic signs of pathology, as shown in Figure 5.

## 6 Discussion

Whilst not the primary objective in this case, cone‐beam computed tomography (CBCT) was fundamental in confirming the incidental finding of the presence of the fusion between the maxillary right central incisor and adjacent supernumerary toot. Current guidelines support the use of CBCT where two‐dimensional imaging fails to provide sufficient diagnostic information.

This highlights the limitations of clinical assessment and two‐dimensional radiography when evaluating the relationship between supernumerary teeth and adjacent permanent teeth. Information garnered from the CBCT was essential for surgical planning, risk assessment and patient discussion, accurately reflecting the intra‐operative findings.

Recognition of the fusion transformed what initially appeared to be a relatively routine supernumerary extraction into a more complex case requiring multidisciplinary planning. Surgical removal of the supernumerary carried a risk of damage to, or loss of, the adjacent permanent maxillary central incisor. Given the strategic importance of the maxillary central incisors in terms of facial aesthetics, function and psychosocial wellbeing, preservation became a primary treatment objective.

The nature of the anomaly in this case introduces a sub gingival area protected from manual cleaning, susceptible to potential pathology including periodontal disease and caries. Beyond impeding orthodontic movement, other complications of leaving the teeth in situ are root resorption and cystic change, although both are unlikely given the patient's age and stage of root development [10]. The surgical separation itself also carried a tangible risk of devitalization or loss of the maxillary right central incisor due to reduced palatal bone following osteotomy.

It was decided to remain in a round archwire in preparation for the surgery, to avoid unnecessary root torque on the maxillary right central incisor and therefore lessen the risk of potential iatrogenic damage. Contingency planning and pre‐operative discussion of the risks involved was therefore crucial to ensure valid consent, assisted by the use of CBCT imaging. This was particularly important due to the patient's high lip line, which heightened the risk of an unfavorable aesthetic result with prosthetic replacement. The patient's age limited replacement options as implant placement in adolescence is usually deferred until growth completion due to the risk of changes in position of the implant as the jaws grow [11]. There are several examples of successful sectioning of fused teeth in the literature, using various methods including rotary hand‐pieces and lasers [12, 13]. Following successful separation, risks highlighted in the literature include dentin hypersensitivity, pulpitis and downstream resorption and the potential need for further treatment including root canal treatment or even extraction [14, 15]. Therefore, the surgical and postoperative complications were weighed against the risks of leaving the supernumeraries in situ before proceeding. The presurgical orthodontic splinting and subsequent successful surgery advocates for such sequencing of treatment when there are concerns regarding compromised bone support. These considerations are particularly relevant when subsequent orthodontic tooth movement is planned. Furthermore, regular assessment of pulpal vitality, periodontal health and radiographic appearance should also form part of the long‐term follow‐up protocol.

## 7 Conclusion

This case report highlights the diagnostic value of dental CBCT in identifying a fusion that was not clinically apparent or expected. Early recognition enabled a coordinated approach and this case report offers a framework for managing similar cases in the future. It demonstrates how rare dental developmental anomalies such as fusions and supernumerary teeth often require a collaborative, multidisciplinary treatment plan to ensure effective management. The pre‐surgical fixed appliance placement demonstrates how orthodontics are useful not only for alignment but also for anchorage and stabilization in complex surgical procedures. The thorough examinations carried out by the various disciplines emphasize the need for detailed pre‐surgical planning and a multidisciplinary team approach, which is vital with regards to both contingency planning and consent.

## Author Contributions

**Andrew Irvine:** writing – original draft, writing – review and editing. **Saarah Juman:** writing – review and editing, supervision. **John Marley:** supervision, writing – review and editing. **Patrick Fee:** writing – review and editing, supervision. **Alistair McNeill:** supervision, writing – review and editing. **Chris Johnston:** writing – review and editing, supervision.

## Funding

The authors have nothing to report.

## Consent

Written informed consent was obtained from the patient for their anonymized information to be published in this article.

## Conflicts of Interest

The authors declare no conflicts of interest.

## Data Availability

The authors have nothing to report.
